# Evaluation of sialometry and minor salivary gland biopsy in classification of Sjögren's Syndrome patients

**DOI:** 10.1016/S1808-8694(15)31334-3

**Published:** 2015-10-20

**Authors:** Bianca Maria Liquidato, Ivo Bussoloti Filho

**Affiliations:** ^1^ Doutora em Otorrinolaringologia pela F.C.M. Santa Casa de São Paulo (Prof. Instrutora do Depto. de Morfologia da F.C.M. Santa Casa de São Paulo); ^2^ Ph.D. in Otorhinolaryngology, UNIFESP; Study carried out at Department of Otorhinolaryngology, Faculdade de Ciências Médicas da Santa Casa de São Paulo

**Keywords:** Sjögren's syndrome, xerostomia, salivary glands, saliva, diagnosis

## Abstract

**S**jögren's Syndrome is an autoimmune disease of the exocrine glands, mainly salivary and lachrymal glands. There is no gold standard test for diagnosis. **Aim**: evaluation of the importance of minor salivary gland biopsy and sialometry, isolated or associated, as methods for classification of Sjögren's Syndrome. **Study design**: Transversal cohort. **Patients and Method**: Seventy-two patients that reported dry mouth from January 1997 to September 2003 were investigated and classified, based on the established criteria. Non-stimulated sialometry was performed by the swab technique. Histopathology exams were evaluated for the presence of inflammatory focus. **Results**: Non-stimulated sialometry and minor salivary gland biopsy presented different sensitivities for primary Sjögren's Syndrome and for secondary Sjögren's Syndrome. Focal sialadenitis with higher focus score was characteristic of primary Sjögren's Syndrome. Biopsy and sialometry were compared and it was observed that specificity and positive predictive value of biopsy were higher. Comparing biopsy and biopsy associated with sialometry, it was observed that biopsy had higher sensitivity and negative predictive value. Specificity of biopsy associated with sialometry was higher. Comparing sialometry and biopsy associated with sialometry, it was observed that biopsy associated with sialometry presented higher positive predictive value and higher specificity. Sialometry's sensitivity was higher. **Conclusions: S**ialometry and biopsy tests presented different performances in primary Sjögren's Syndrome and secondary Sjögren's Syndrome; the positivity of the association of both tests increases the specificity for Sjögren's Syndrome (95%).

## INTRODUCTION

Sjögren's Syndrome is an autoimmune affection of the exocrine glands that involves salivary and lachrymal glands. The clinical spectrum is broad and it may range from reduction of lachrymation and xerostomia to joint, pulmonary and renal impairment. Primary Sjögren's Syndrome takes placed isolated and secondary Sjögren's Syndrome (SS) manifests concomitantly with other autoimmune diseases, especially lupus and rheumatoid arthritis.^1^

SS affects mainly female patients with prevalence of 9 women to 1 man, and age of symptom onset is about 40 to 45 years. It is rare in children^2^. Mean age at diagnosis is 50 years^3^. Its real prevalence is hard to be defined as a result of the difficulty to diagnose it, but it is estimated to be 1% to 3% of the population^1^.

There is a tissue inflammatory infiltrate that affects target organs, normally comprised in most cases (60 to 70%) by T CD4 lymphocytes and by a minority of lymphocytes B. However, there is hyperreactivity of B cells with mono or polyclonal production of IgM or IgG immunoglobulins. Epithelial cells of salivary glands express cytokines such as interleukin 1β, interleukin 6 and TNFα. Autoimmune response is directed to rhibonucleoproteins Ro/SS-A with two protein chains of 52 and 60 kDa (cytoplasm) and La/SS-B (nuclear) of 48 kDa. The mechanism that may be responsible for the autoimmune process is apoptosis of epithelial cell, in which intracellular auto-antigens are exposed to the immune system on the surface of the apoptotic blisters^4^.

More than one third of patients have systemic manifestations that can include vasculitis, cryoglobulinemia, autoimmune hepatitis, pulmonary fibrosis, central nervous system impairment, renal tubular acidosis, B cell lymphoma and multiple myeloma1., 2., 4.. Thus, the appropriate diagnosis of the manifestation is important to provide symptom relief^3^ but also clinical follow up of the possible complications, considering that those are late events in the syndrome^5^.

Saliva has an important role in gustation, mastication, swallowing and speech, as a result of the lubricant role in the oral cavity; in addition, many enzymes that comprise saliva act as antimicrobial agent^6^. The reduction of saliva leads to repercussions in swallowing and speech, with complications such as erythematous candidiasis and tooth decays.

Many studies tried to define classification criteria for Sjögren's Syndrome considering that there is no final diagnostic exam. Such criteria started to be employed in different services, hindering the interpretation of international literature. A multicenter study performed in 12 countries, guided by The European Community Study Group on Diagnostic Criteria for Sjögren's Syndrome, tried to define reliable criteria to standardize diagnoses1., 7., 8.. For primary Sjögren's Syndrome, there should be 4 positive criteria out of 6. For secondary Sjögren's Syndrome, the presence of 3 specific criteria is enough.

The sialometry technique used in the European Community Study Group on Diagnostic Criteria for Sjögren's Syndrome is a collection of non-stimulated saliva for 15 minutes and 5-minute collection after chewing paraffin to ensure saliva stimulation. Non-stimulated sialometry is preferred because it has less influence of the age of subjects1., 7..

Positivity of autoantibodies ranges at about 70% for anti-SSA and 60% for anti-SSB according to the methods used for detection.

Minor salivary gland biopsy is the most accurate exam^8^, but it is not a sine qua non criterion for the diagnosis of Sjögren's Syndrome and together with the autoantibodies, they are considered the most specific exams. In past years, many studies have been directed to non-invasive methods for the diagnosis of Sjögren's Syndrome especially by the dosage of substances present in the inflammatory process of saliva and tears. However, there are still no definite studies that validate their use as diagnostic criterion. Other imaging exams, such as magnetic resonance sialography, have been studied as non-invasive alternatives for traditional sialography, trying to avoid the possible complications that include duct trauma, pain upon contrast injection and allergic reaction.

The participation of the Otorhinolaryngologist is essential for the diagnosis, not only to perform minor salivary gland biopsy but also to assess gland involvement, always trying to perform less invasive exams. The technique of sialometry with cotton weighing that has been used in our center^9^ has proven to be easy and practical to perform compared to other methods and it is less invasive than parotid sialography.

In daily practice, the diagnosis of Sjögren's Syndrome can be made based only on clinical impressions, but to include a patient in scientific studies, it is necessary to have diagnostic confirmation through the criteria obtained from the clinical history and objective complementary exams10., 11..

The present study aimed at assessing the role of minor salivary gland biopsy and sialometry, either isolated or associated, as methods used to classify Sjögren's Syndrome based on the criteria defined by the European Community Study Group on Diagnostic Criteria for Sjögren's Syndrome1., 7., 8., 11., 12..

## MATERIAL AND METHOD

All 72 patients complaining of dry mouth that came to the Department of Otorhinolaryngology, Santa Casa de São Paulo, from January 1997 to September 2003, were clinically assessed at the Ambulatory of Stomathology and they were submitted to diagnostic investigation and classification based on the criteria defined by the American-European Consensus^11^ (Chart 1).

**Chart 1:** Criteria for classification of Sjögren's Syndrome.

I. Ocular symptoms, positive response for at least one of the following questions: 1. Have you felt your eyes dry for the past 3 months? 2. Do you have a recurrent feeling of sand in the eyes? 3. Do you use tear substitutes more than 3 times a day? II. Oral symptoms, positive response to at least one of the following questions: 1. Have you felt your mouth dry for the past 3 months? 2. Have you had recurrent or persistent increase in salivary glands in adult life? 3. Do you normally drink liquids to help you swallow dry foods? III. Ocular impairment signs, positive results in one of the two following tests: • Schirmer I Test (< or = 5 mm within 5 min); • Rose Bengal or other dye test (> or = 4). IV. Histopathology: presence of 1 or more foci (agglomerate of 50 or more inflammatory cells) by 4mm2 of gland tissue in minor salivary gland biopsy. V. Salivary gland involvement, positive result for one of the following diagnostic tests: • Sialometry with total non-stimulated flow < or = 1.5 ml within 15 minutes; • Parotid Sialography showing diffuse sialectasia, without evidence of major duct obstruction; • Salivary scintigraphy with delay in recording, reduction in concentration and/or delay in tracing secretion. VI. Auto-antibodies, presence of one or both: Anti -Ro antibodies (SS-A) or anti-La antibodies (SS-B).

Source: Vitali et al. (2002).

These patients were divided into 2 groups: the group that did not present Sjögren's Syndrome (NSS) and the group that had Sjögren's Syndrome (SS). The group that had SS was divided into 2 subgroups: with primary Sjögren's Syndrome (SSp) and secondary Sjögren's Syndrome (SSsec).

To classify patients with primary Sjögren's Syndrome we required the presence of 4 out of 6 items and item 4 (histopathology) or 6 (auto-antibodies) had necessarily to be present. As to classification of patients with secondary Sjögren's Syndrome, it required the presence of item 1 or item 2 plus 2 other items numbered 3, 4 and 5 (Chart 1).

We considered as exclusion criteria for classification of Sjögren's Syndrome: previous head and neck radiotherapy; Hepatitis C; AIDS; preexisting lymphoma; sarcoidosis; graft versus host disease; use of anti-cholinergic drugs. We excluded from the study one patient that had Wegener's granulomatosis.

The progression of the disease was considered as the period between onset of symptoms and first visit at the Ambulatory of Stomathology, Department of Otorhinolaryngology.

Non-stimulated sialometry was performed with saliva collection technique using two cotton balls that had been previously weighted with the 80ml universal collection container, using a regular digital scale. Patients were instructed to swallow all saliva they had in the oral cavity and then the cotton balls were placed on the floor of the mouth, close to the gingival border, where they remained for 2 minutes. After this time, the set was weighted again. The difference in weight was transformed from g/min directly into ml/minute and any sialometry that had value below 0.1 ml/minute was considered abnormal.

The technique of minor salivary gland biopsy, chosen as the preferred one, was performed with a horizontal incision on the mucous face of the lower lip parallel to the lip vermillion, with removal of 4 to 6 minor salivary glands, but no removal of lip mucosa. This material was placed in containers with 10% formol, processed with paraffin inclusion, submitted to sections of rotation microtome, producing 3-micrometer thick sections. Sections were stained with hematoxylin-eosin (HE) and submitted to histology analysis. The slides were later reviewed using a Zeiss microscope model Axioskop 40, coupled to a computer with Intel Pentium III processor and supported by software Axiovision 3.1, to limit the gland area tissue. In this review, the glandular tissue was measured using a cursor in different fields, with 50X enlargement, and histopathological analyses were classified based on presence of inflammatory infiltrate and presence of inflammatory foci, which consisted on 50 lymphocytarian cell agglomerates. Foci count in the total area of glandular tissue in each exam was corrected in each one to correspond to 4mm^2^. Slides with the respective areas measured in glandular tissue were photographed, amounting to 280 photos.

**Figure 1:**
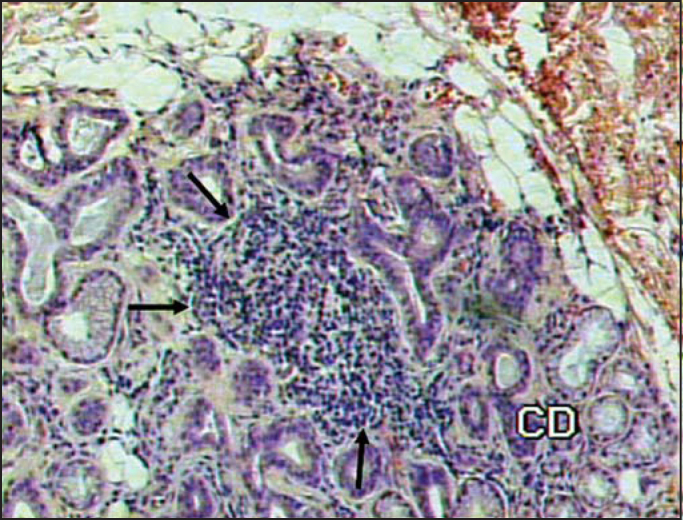
Presence of inflammatory focus amidst glandular tissue, indicated by the arrows (HE 100X).

**Figure 2:**
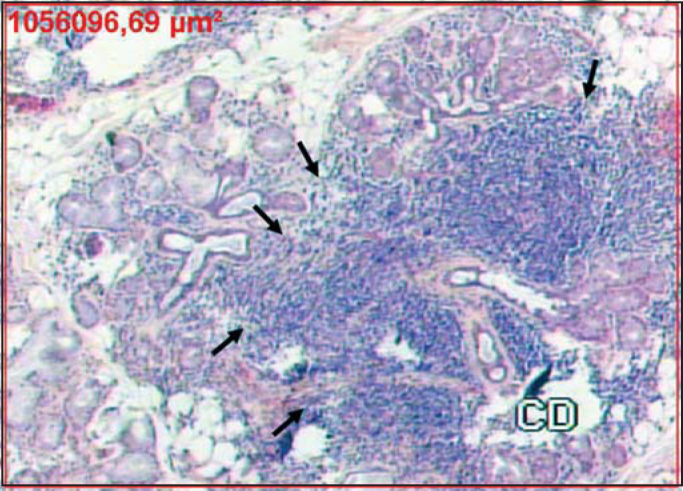
Confluent inflammatory foci indicated by the arrows that replace the glandular tissue (HE 50X). The red rectangle corresponds to area measurement.

Histopathological findings were graded as follows: normal gland; mild inflammatory process; moderate inflammatory process; severe inflammatory process, and presence of inflammatory foci (Figures 1 and 2).

Based on the selected Statistical Method, we used minimum, maximum, median, mean and standard deviation values to describe the quantitative variables and Wilcoxon test for independent samples and Kruskal-Wallis test for the comparison between 2 groups and more than 2 groups. In the case in which we detected statistically significant difference between the groups, we performed multiple comparisons using Dunn test to identify the differences.

Pearson correlation coefficient was used to study the association between number of foci and duration of disease progression.

We calculated sensitivity, specificity, positive and negative predictive vales and accuracy of biopsy, sialometry and the combinations of both results. The comparison of such indexes between the exams was made using linear models for categorized data, using Wald's statistics. The adopted level of significance was 5%.

The study was approved by the Ethics Committee of the Institution.

## RESULTS

Out of 72 patients with complaints of xerostomia, 26 (36.1%) were classified as having Sjögren's Syndrome, based on the presented criteria, and 46 (63.9%) presented different etiological diagnoses and were classified as non-Sjögren Syndrome (NSS).

Patients in group SS were divided into 2 subgroups: 17 (65.4%) presented primary Sjögren's Syndrome and 9 (34.6%), presented secondary Sjögren's Syndrome (SSsec).

Out of 46 cases in the NSS group, 7 (15.2%) were submitted to head and neck radiotherapy; 3 (6.5%) had had diagnosis of diabetes; 3 (6.5%) presented positive serology for hepatitis C; 12 (26.1%) made use of xerostomia drugs; 6 (13.1%) had chronic sialadenitis; 1 (2.2%) presented Wegener's granulomatosis; 4 (8.7%) met 4 out of 6 criteria but did not present histopathological diagnosis or positive auto-antibodies, and 10 cases (21.7%) did not present definite cause (Figure 10). Out of the patients without definite cause, some are still being investigated and other were lost for the follow up.

Among the 9 cases of subgroup SSsec, 7 (77.8%) were secondary to rheumatoid arthritis; 1 (11.1%) was secondary to lupus; and 1 (11.1%) was secondary to sclerodermia.

As to time of disease progression, the groups presented: NSS with median 2.00 years (minimum of 0.08 and maximum of 18.00); SSp with median of 4.00 years (minimum of 0.25 and maximum of 13.00); and SSsec with median of 3.00 years (minimum of 0,50 and maximum of 7,00), there was no difference between the groups (p = 0.0717). When the analysis was made with the grouping of subgroups SSp and SSsec, the comparison of group SS with median of 3.00 years (minimum of 0.25 and maximum of 13.00) with group NSS, showed difference between the two groups (p = 0.0369).

Analyzing the results of biopsy in relation to number of inflammatory foci in the groups, we observed that: in relation to 37 NSS cases, median of 0.00 (minimum of 0.00 and maximum of 6.00); in 17 cases SSp, median of 5.20 (minimum of 0.00 and maximum of 12.00); and in 8 cases SSsec, median of 0.00 (minimum of 0.00 and maximum of 5.90). The comparison between the groups showed differences relative to number of foci (p < 0.0001).

Still in relation to number of foci in the biopsy, groups NSS, SSp and SSsec were compared taking into account the results of sialometry. When sialometry was abnormal, the test was considered positive (+) and when normal, it was considered negative (-). Kruskal-Wallis test showed significant difference (p = 0.0001) in the comparison of number of foci in all groups, simultaneously. Multiple comparisons were made to identify the differences, represented by different letters (Table 1). Groups with the same letters presented results without significant differences. Pearson correlation test was used to correlate the number of biopsy foci with time of progression, but we did not observe significant results (r = 0.17330; p = 0.1780).

**Table 1:** Comparison of number of foci in groups NSS, SSp and SSsec with results of sialometry.

Group	Sialometry	n	Minimum	Maximum	Median	Mean	p	Result
NSS	+	16	0.00	4.80	0.00	22.8		A
NSS	-	21	0.00	6.00	0.00	24.5		A
SSp	+	12	0.00	9.80	5.75	50.3	0,0001	C
SSp	-	5	0.00	12.00	4.10	45.1		C
SSsec	+	4	0.00	3.60	0.00	25.8		A
SSsec	-	4	0.00	5.90	2.25	35.3		B

(+) represents abnormal result of sialometry

(-) represent normal result of sialometry

A, B and C: groups with the same letters do not present significant differences

For biopsy, sialometry and association of the two criteria - positive biopsy and sialometry we calculated sensitivity, specificity, positive and negative predictive values and accuracy in relation to groups NSS, SSp and SSsec.

To compare indexes of sensitivity, specificity, positive predictive value, negative predictive value and accuracy, subgroups SSp and SSsec were gathered and represented by SS (Table 2).

**Table 2:** Values of sensitivity, specificity, positive predictive value (VP+), negative predictive value (VP-) and accuracy calculated for biopsy, sialometry and positive biopsy and sialometry in groups with primary Sjögren's Syndrome (SSp), secondary Sjögren's Syndrome (SSsec), both together (SS) and non-Sjögren Syndrome.

%	Biopsy	Sialometry	Biopsy and sialometry
Sensitivity SSp	88.2	70.6	64.7
Sensitivity SSsec	37.5	44.4	11.1
Sensitivity SS	72.0	61.5	46.2
Specificity NSS	83.8	52.2	95.0
VP + SSp	62.5	31.6	78.6
VP + SSsec	12.5	10.5	7.1
VP + SS	75.0	42.1	85.7
VP -	81.6	70.6	73.1
Accuracy	79.0	55.6	75.8

We compared positive predictive value of biopsy and sialometry with findings of significant differences between them (p = 0.0036), and biopsy value was higher than sialometry value. There was no difference between negative predictive values (p = 0.0997) and sensitivity (p = 0.5237) of biopsy and sialometry. Comparing specificity, we observed that specificity of biopsy was higher than that of sialometry (p = 0.0106).

Upon comparing the positive predictive value of biopsy and biopsy associated with sialometry, we evidenced that there were no significant differences (p = 0.1553). However, negative predictive values of them both (p = 0.0129), as well as sensitivity (p = 0.0051) are different and higher than for biopsy. Specificity of biopsy and biopsy associated with sialometry presented significant difference (p = 0.0350), higher for biopsy associated with sialometry.

Upon comparing positive predictive value of sialometry and biopsy associated with sialometry, we observed difference between them (p < 0.0001), where value of biopsy associated with sialometry was higher. However, negative predictive value of sialometry and biopsy associated with sialometry showed no significant differences (p = 0.5662). Sensitivity of sialometry was higher (p = 0.0304) than biopsy associated with sialometry, and specificity of biopsy associated with sialometry was higher than the one for sialometry alone (p < 0.0001).

## DISCUSSION

The attempt to define criteria to make the diagnosis of patients with Sjögren's Syndrome has occupied the scientific literature for many decades. It is agreed that maybe the only reliable gold standard is the clinical judgment of an experienced physician^12^. Owing to the fact that there is no gold standard for the diagnosis, what we try to do is to set criteria to do so.

Classifications initially include a series of criteria and according to the number of criteria met by the patient he/she would be classified as having Sjögren's Syndrome10., 13., which generated some uncertainty for patients and difficulty for researchers to enroll patients in scientific trials.

Another difficulty found for the definition of the classification criteria is the small number of patients with Sjögren's Syndrome, which was solved by the conduction of multicenter studies, involving different countries, conducting exams and applying questionnaires in groups, controlling with over 200 cases1., 7..

Owing to the fact that it is the Otorhinolaryngologist that should get to know and identify diseases that affect the salivary glands and those that can interfere in the production of saliva, we tried to identify among those patients that came to the Ambulatory of Stomathology, Department of Otorhinolaryngology, those that really presented Sjögren's Syndrome. To that end, the criteria set by the European Community Study Group on Diagnostic Criteria for Sjögren's Syndrome1., 7., 8., 11., based on the descriptive method was used. To assess ocular impairment, Schirmer test is more frequently used than Rose-Bengal owing to the facility to make and the familiarity with the test technique by the Otorhinolaryngologist, which uses it to assess patients with facial paralysis. In the investigation of salivary gland involvement, scintigraphy and sialometry were more used than parotid sialography, also owing to easy execution, but specially because they are less invasive methods.

Still regarding sialometry, the preferred technique was salivary collection with previously weighted cotton^9^, because of its practical approach in view of the other techniques that involve volunteer actions of patients such as spitting or collecting saliva from the mouth.

As to sensitivity and specificity of the test described in the literature, biopsy presented sensitivity of 82.4% and specificity of 86.2%, and non-stimulated sialometry presented sensitivity of 56.1% and specificity of 80.7%^7^. In the present results, we observed biopsy with lower sensitivity, 72%, and similar specificity, 83.8%, and sialometry with higher sensitivity, 61.5%, and lower specificity, 52.2%. Probably, these differences are caused by size of sample, but the biopsy maintained the specificity even with smaller sample. Biopsy was the test with higher accuracy, 89%^8^ and in the present results we presented slightly lower accuracy, 79%.

We observed that the association of two positive criteria, sialometry and biopsy, presented very high specificity - 95%, higher than isolated biopsy. Such fact has major importance thinking about the criteria for the classification of patients in research studies, in which high specificity will increase the strict criteria for inclusion of cases.

The classification of patients in this study was based on modifications introduced by the American-European Consensus, that is, the mandatory presence of biopsy or the presence of auto-antibodies, among the four international criteria for patients to be considered with primary Sjögren's Syndrome. Thus, to classify patients in group SSp, we considered as mandatory the presence of biopsy or positive auto-antibodies^11^. This topic has been object of controversy in the international literature^14^. As a result, 4 patients in the study, which would be diagnosed as Sjögren's Syndrome, were reclassified as NSS (19%), because they presented 4 positive criteria, but they did not present biopsy or positive auto-antibodies. In the literature, this reclassification index was higher^15^.

Even though they are not part of the exclusion criteria for Sjögren's Syndrome^11^, we decided to exclude one case that met all criteria for primary Sjögren's Syndrome, but the patient presented Wegener's granulomatosis. As already described^16^, Wegener's granulomatosis may be present with salivary gland impairment, with increase in volume, possibility of functional impairment and histopathological exam, which is compatible with Sjögren's Syndrome.

There is multiplicity of sialometry techniques, and as a result, many nomenclature systems, such as salivary flow measurements at rest, or basal flow or non-stimulated flow. There are also many techniques of stimulation, and in addition, many variations in relation to collection time and normal range parameters. In the present study, we considered only the non-stimulated sialometry values as defined by the classification criteria. Stimulated sialometry has a role in the assessment of glandular reserve and, thus, the indication of the best choice in relation to hyposalivation treatment. If there is reserve, we should make an attempt to stimulate the gland; if not, we should prefer the use of saliva substitutes.

As to non-stimulated salivary flow, we can use it as drainage techniques - spit, suction and swab, with the use of cotton^17^. The adoption of swab technique^9^ proved to be practical, of low cost, easy to measure and provided autonomy to patients' acts, such as for example, spitting. It seems to be important when we assess elderly patients or those with some degree of motor impairment.

Dry mouth complaint - xerostomia, is not always correlated with objective signals of hyposalivation18., 19., 20., 21., 22.. In our study, we considered it difficult to define a correlation between dry mouth complaint and abnormal sialometry, but in some cases, subjects with xerostomia present sialometry within the normal range. It may occur by a reduction of saliva flow, which is significant for the patients, but whose values were within the normal range, or still by reducing the production of mucin, an abnormality in qualitative composition of saliva. Conversely, there are subjects with very low flows in sialometry, many times with values that leave no doubts.

As to the idea that the elderly presented higher prevalence of dry mouth, in fact, the elderly did not present dry mouth owing to aging per se, but due to higher prevalence of associated diseases and use of drugs23., 24., 25.. In the present study, 6.5% of the patients NSS presented diabetes as cause of symptoms and 26.1% used medications that caused xerostomia.

As to normal range criteria, the value of 0.1 ml/min corresponds to the adopted classification criteria of Sjögren's Syndrome and it was adopted by this study19., 21.. However, this value was also widely discussed by other publications22., 25., 26..

In this study, non-stimulated sialometry presented SSp sensitivity of 70.6% and SSsec of 44.4%. Specificity was 52.2%. We observed that test performance was very different between SSp and SSsec. When groups SSp and SSsec were grouped in SS, sensitivity was 61.5%, somewhat higher than what was related in the literature, and specificity was maintained in 52.2%, lower than the one described in the literature - 80.7%^7^. Such differences may also be explained by size of sample. Positive predictive value was 42.1% and negative predictive value (PV) was 70.6%, or in other words, there is higher likelihood of the subject not having Sjögren's Syndrome when sialometry is negative. Accuracy was of 55.6%.

Minor salivary gland biopsy was maintained as an alternative to assess salivary gland impairment in Sjögren's Syndrome. Morbidity is very low compared to major salivary gland biopsy, especially with the technical modification of isolated incision, removing the glands through the incision^27^, differently from what was used, which removed a section of the mucosa^28^.

Even though it is considered the highest accuracy among the classification criteria, minor salivary gland biopsy is also surrounded by controversy, specially concerning the number of foci present in it that is suggestive of Sjögren's Syndrome. Some authors stated that one inflammatory foci in 4mm2 of gland tissue would be suggestive of Sjögren's Syndrome1., 28., whereas others stated that it would be necessary to have more than one 4mm2 focus for the diagnosis of salivary impairment in Sjögren's Syndrome29., 30., 31.. Others questioned the value of the biopsy stating that similar picture could be present in other diseases^32^.

Based on the studies published by the European Community Study Group, in special in 1993 and 19941., 7., in which minor salivary gland biopsy was validated and tested in terms of sensitivity and specificity, considering as limit the presence of 1 focus and also more than one focus, the authors demonstrated that gain in specificity was minor compared to loss in sensitivity when we considered more than one focus. Thus, there is some tendency in adopting this limit to standardize scientific studies. In the present study we considered the positivity of histopathological exams with the presence of 1 or more 4mm2 foci of glandular tissue.

During the review of slides of patients NSS and SS, we observed different types of inflammatory infiltrate and tried to grade them as mild, intermediate and severe^30^; we also counted the inflammatory foci, which is essential to define whether the biopsy was suggestive or not of Sjögren's Syndrome.

We observed significant difference between medians of number of foci in groups NSS (0.00), SSp (5.20) and SSsec (0.00), that is, in fact focal sialodenitis with higher number of foci was characteristic of primary Sjögren's Syndrome, confirming also the statement that patients with secondary Sjögren's Syndrome presented fewer foci in biopsies^7^.

The fact that the biopsy is considered a mandatory criterion for classification of patients with primary Sjögren's syndrome and not mandatory for the classification of secondary Sjögren's syndrome may be owed to the same fact that indirectly reflects the difference existing in both conditions, that is, they are different laboratory and histopathological clinical manifestations.

The comparison of number of foci in groups NSS, SSp and SSsec, subdivided according to the results of sialometry, either abnormal or normal, demonstrated in Table 1, showed that there is no difference in the results obtained in groups NSS with abnormal sialometry and normal sialometry, and SSp with abnormal sialometry and normal sialometry. SSp group continued to have significantly more foci, regardless of the results of sialometry. There was difference in the group SSsec, in which those that presented affected sialometry had results similar to those in group NSS. Thus, we can observe that salivary flow affections represented by abnormal sialometry did not have any correlation with number of foci presented in the biopsy.

Still concerning number of foci, we did not observe any correlation between higher number of foci and higher disease progression time, which makes us consider that the severity of the condition and the impairment of salivary gland are independent from duration of the disease, or in other words, a subject that presented Sjögren's syndrome for little time could have had more impairment than a subject with Sjögren's syndrome for many years, which is controlled and without sudden episodes.

As to role of minor salivary gland biopsy as diagnostic criteria, we observed that it presented sensitivity of 88.2% for SSp, quite different from the sensitivity for SSsec, which was 37.5%, which once again reflects a very different profile from the test in each condition. Specificity was 83.8%. When groups SSp and SSsec were brought together in SS, we confirmed test sensitivity of 72.0%, somewhat lower than what was described in the literature^7^, of 82.4%, and specificity was maintained at 83.8%, quite similar to that described in the same study, of 86.2%. It shows that test specificity was maintained, Positive PV was 75% and Negative PV was 81.6%, showing that a subject would have 75% likelihood of having Sjögren's syndrome when biopsy was positive and 81.6% likelihood of not having Sjögren's syndrome when biopsy was negative. Accuracy was 79.0%.

When tests were analyzed together, that is, positive biopsy and sialometry, sensitivity dropped to 46.2%; however, specificity went up to 95.0%. Positive PV was 85.7% and Negative PV was 73.1%. Accuracy was 75.8%. We detected expressive increase in specificity for this association.

Upon comparing biopsy and sialometry, we could observe that specificity and Positive PV of biopsy were higher and there was no difference between sensitivity and negative PV. Thus, if necessary to use these tests for screening purposes, both isolated biopsy and sialometry may be used owing to the fact that they do not have any difference in sensitivity.

When the comparison was made between the biopsy and biopsy associated with sialometry, we observed that biopsy had higher sensitivity and higher negative PV. Positive PV did not present differences. Specificity of biopsy associated with sialometry was higher.

Upon comparing sialometry and biopsy associated with sialometry, we could observe that the association had higher positive PV and higher specificity. Sensitivity of sialometry was higher. There were no statistically significant differences between negative PV. Once again, specificity of biopsy plus sialometry was higher than for the isolated tests.

Therefore, we can notice that the positive results of biopsy plus sialometry increased specificity for Sjögren's syndrome, differently from the situation in which they are considered separately. We understand that loss of sensitivity may be compensated by the use of other criteria that are not object of the present study.

Owing to the fact that there has been no difference between positive PV of biopsy and biopsy plus sialometry, and that positive PV of biopsy plus sialometry is higher than positive PV for sialometry alone, we can state that subjects have a likelihood of 85.7% of having Sjögren's syndrome when both exams are positive, a likelihood that is quite high.

Thus, if there are any limitations concerning the exams to be performed to classify a patient, the combination of positive biopsy and sialometry shows high chances of making the right classification.

## CONCLUSION

Based on the observations of this study, we concluded that:

Tests of sialometry and biopsy have different performances in patients with primary and secondary Sjögren's Syndrome. Sensitivity and positive predictive values were higher for primary Sjögren Syndrome, both isolated and together. The number of biopsy foci was also higher in primary Sjögren's Syndrome.

Sialometry performed with cotton technique presented sensitivity of 61.5% and negative predictive value of 70.6%.

Biopsy presented specificity of 83.8% and positive predictive value of 75%.

Positive response for both criteria together increases the specificity for Sjögren's Syndrome (95%) when compared to the isolated tests, which is important for the classification of research studies. The positive predictive value of both criteria together was 85.7%.

## References

[1] Vitali C, Bombardieri S, Moutsopoulos HM, Balestrieri G, Bencivelli W, Bernstein RM (1993). Preliminary criteria for Sjögren's Syndrome. Results of a prospective concerted action supported by the European Community.. Arthritis Rheum.

[2] Bartunková J, Sedivá A, Vencovsky J, Tesar V. (1999). Primary Sjögren's syndrome in children and adolescents proposal for diagnostic criteria.. Clin Exp Rheumatol.

[3] Bell M, Askari A, Bookman A, Frydrych S, Lamont J, Mccomb J (1999). Sjögren's syndrome: a critical review of clinical management.. J Rheumatol.

[4] Tapinos NI, Polihronis M, Tzioufas AG, Moutsopoulos HM. (1999). Sjögren's Syndrome. Autoimmune epithelitis.. Adv Exp Med Biol.

[5] Skopouli FN, Dafni U, Ioannidis JPA, Moutsopoulos HM. (2000). Clinical evolution, and morbidity and mortality of primary Sjögren's syndrome.. Semin Arthritis Rheum.

[6] Scully C. (2001). The role of saliva in oral health problems.. Practioner.

[7] Vitali C, Moutsopoulos HM, Bombardieri S. (1994). The European Community Study Group on Diagnostic Criteria for Sjögren's Syndrome. Sensitivity and specificity of tests for ocular and oral involvement in Sjögren's Syndrome.. Ann Rheum Dis.

[8] Vitali C, Bombardieri S, Moutsopoulos HM, Coll J, Gerli R, Hatron PY. (1996). The European Community Study Group On Diagnostic Criteria For Sjögren's Syndrome. Assessment of the European classification criteria for Sjögren's syndrome in a series of clinically defined cases: results of a prospective multicentre study.. Ann Rheum Dis.

[9] Pupo DB, Bussoloti Filho I, Liquidato BM, Korn GP. (2002). Proposta de um método prático de sialometria.. Rev Bras Otorrinolaringol.

[10] Manthorpe R. (2001). New criteria for diagnosing Sjögren's syndrome: a step forward. Scand J Rheumatol.

[11] Vitali C, Bombardieri S, Jonsson R, Moutsopoulos HM, Alexander EL, Carsons SE. (2002). The european study group on classification criteria for Sjögren's syndrome. Classification criteria for Sjögren's syndrome: a revised version of the European criteria proposed by the American-European Consensus Group.. Ann Rheum Dis.

[12] Vitali C, Bombardieri S. (1998). The diagnosis of Sjögren's syndrome: definition and validation of classification criteria for this disorder.. Ann Med Interne.

[13] Fox RI, Robinson CA, Curd JG, Kozin F, Howell FV. (1986). Sjögren's Syndrome: Proposed criteria for classification.. Arthritis Rheum.

[14] Manthorpe R. (2002). Sjögren's syndrome criteria. American-European and Japanese Groups' criteria compared and contrasted.. Ann Rheum Dis.

[15] Brun JG, Madland TM, Gjesdal CB, Bertelsen LT. (2002). Sjögren's syndrome in an out-patient clinic: classification of patients according to the preliminary European criteria and the proposed modified European criteria.. Rheumatology.

[16] Böttinger EP, Niles JL, Collins AB, Mccluskey RT, Arnaout MA. (1992). Antineutrophil cytoplasmic autoantibody-associated vasculitis presenting as Sjögren's syndrome.. Arthritis Rheum.

[17] Navazesh M, Chistensen CM. (1982). A comparison of whole mouth resting and stimulated salivary measurement procedures.. J Dent Res.

[18] Fox PC, Busch KA, Baum BJ. (1987). Subjective reports of xerostomia and objective measures of salivary gland performance.. J Am Dent Assoc.

[19] Sreebny LM, Valdini A. (1988). Xerostomia. Part I: Relationship to the other oral symptoms and salivary gland hypofunction.. Oral Surg Oral Med Oral Pathol.

[20] Wang SL, Zhao ZT, Li J, Zhu XZ, Dong H, Zhang YG. (1998). Investigation of the clinical value of total saliva flow rates.. Arch Oral Biol.

[21] Hay EM, Thomas E, Pal B, Hajeer A, Chambers H, Silman AJ. (1998). Weak association between subjective symptoms of and objective testing for dry eyes and dry mouth: results from a population based study.. Ann Rheum Dis.

[22] Longman LP, Mccracken CFM, Higham SM, Field EA. (2000). The clinical assessment of oral dryness is a significant predictor of salivary gland hipofunction.. Oral dis.

[23] Sreebny LM, Valdini A, Yu A. (1989). Xerostomia. Part II: Relationship to nonoral Symptoms, drugs, and diseases.. Oral Surg Oral Med Oral Pathol.

[24] Baum BJ. (1989). Salivary gland fluid secretion during aging.. J Am Geriatr Soc.

[25] Ship JA, Fox PC, Baum BJ. (1991). How much saliva is enough? “Normal” function defined.. J Am Dent Assoc.

[26] Crockett DN. (1993). Xerostomia: the missing diagnosis. Aust Dent J.

[27] Greenspan JS, Path MRC, Daniels TE, Talal N, Sylvester MD. (1974). The histopathology of Sjögren's syndrome in labial salivary gland biopsies.. Oral Surg.

[28] Chisholm DM, Mason K. (1968). Labial salivary gland biopsy in Sjögren disease.. J Clin Pathol.

[29] Daniels TE. (1984). Labial salivary gland biopsy in Sjögren's syndrome. Assessment as a diagnostic criterion in 362 suspected cases.. Arthritis Rheum.

[30] Daniels TE, Whitcher JP. (1994). Association of patterns of labial salivary gland inflammation with keratoconjunctivitis sicca.. Arthritis Rheum.

[31] Manthorpe R, Benoni C, Jacobsson L, Kirvata Z, Larsson A, Liedholm R (2000). Lower frequency of focal lip sialadenitis (focus score) in smoking patients. Can tobacco diminish the salivary gland involvement as judged by histological examination and anti-SSA/Ro and anti-SSB/La antibodies in Sjögen's syndrome. Ann Rheum Dis.

[32] Lindahl G, Hedfors E. (1989). Lymphocytic infiltrates and epithelial HLA-DR expression in lip salivary glands in connective disease patients lacking sicca: a prospective study.. Br J Rheumatol.

